# Ethyl Pyruvate Inhibits Retinal Pathogenic Neovascularization by Downregulating HMGB1 Expression

**DOI:** 10.1155/2013/245271

**Published:** 2013-11-25

**Authors:** Yun Mi Lee, Junghyun Kim, Kyuhyung Jo, So Dam Shin, Chan-Sik Kim, Eun Jin Sohn, Seon Gi Kim, Jin Sook Kim

**Affiliations:** Korean Medicine Based Herbal Drug Development Group, Herbal Medicine Research Division, Korea Institute of Oriental Medicine (KIOM), 1672 Yuseongdaero, Yuseong-gu, Daejeon 305-811, Republic of Korea

## Abstract

Retinal pathogenic angiogenesis in the eyes is a causative factor in retinopathy of prematurity, diabetic retinopathy, and age-related macular degeneration. This study was designed to examine the pathogenic role of the high-mobility group box-1 (HMGB1) protein and the inhibitory effect of ethyl pyruvate (EP), a well-known antioxidant substance, in retinal pathogenic angiogenesis in mice with oxygen-induced retinopathy (OIR), one of the animal models of proliferative ischemic retinopathy. The OIR mouse model was used for our in vivo studies. The mice were exposed to 75% oxygen from postnatal day 7 (P7) to P11, after which the mice were brought to room air and intraperitoneally injected with EP (50 mg/kg, or 100 mg/kg) for five days. At P17, the mice were perfused with fluorescein isothiocyanate-dextran, and flat-mounted retinas were used to measure nonperfused and neovascular tufts. In OIR mice, an intraperitoneal injection of EP reduced the nonperfused retinal area in the treatment group and significantly reduced the retinal neovascular tufts. In addition, EP inhibited the overexpression of HMGB1 in the retinas of OIR mice. These data suggest that EP could serve as an innovative pharmaceutical agent to prevent retinal neovascularization through inhibiting HMGB1 expression.

## 1. Introduction

Angiogenesis, the formation of new vessels from existing vessels, plays an important role in pathological conditions in various organs [1]. Pathological angiogenesis in the eye is the most common cause of blindness in all age groups. Retinopathy of prematurity (ROP) occurs in children, diabetic retinopathy (DR) in young adults, and age-related macular degeneration (AMD) in the elderly [2]. It is important to understand the mechanism of underlying pathological neovascularization to identify new targets to treat these diseases. Vascular endothelial growth factor (VEGF) is thought to be the major angiogenesis factor in ROP, DR, and AMD [3]. Recently, some evidence indicates that chronic inflammation is also implicated in the pathogenesis of retinal neovascularization [4, 5]. The relationship between chronic inflammation and pathogenic angiogenesis is widely accepted [6]. The high-mobility group box-1 (HMGB1) protein was initially discovered as a nuclear chromatin-binding protein that stabilizes nucleosome formation and facilitates transcription. Necrotic cell death can result in passive leakage of HMGB1 from the cell. HMGB1 can be actively secreted by various cell types, including activated monocytes and macrophages, and endothelial cells, after inflammatory stimuli [7, 8]. Extracellular HMGB1 functions as a proinflammatory cytokine [6, 9] and exhibits angiogenic effects [10, 11]. HMGB1 signals through the receptor for advanced glycation end products (RAGE) leading to the activation of the transcription factor nuclear factor kappa B (NF-*κ*B) and induces the expression of various leukocyte adhesion molecules proinflammatory cytokines, chemokines, and angiogenic factors [6, 9]. In a previous report, increased levels of HMGB1 were found in vitreous samples from patients with proliferative DR [12], and HMGB1 expression was upregulated in the retinas of diabetic mice [13].

Ethyl pyruvate (EP) is derived from pyruvate by the addition of an aliphatic ester group and is more stable and safer than pyruvate in inhibiting the generation of reactive oxygen species (ROS) and inflammation [14]. EP is also a potent HMGB1 inhibitor [15]. Moreover, EP inhibits tumor angiogenesis and intracerebral hemorrhage-induced angiogenesis [16, 17]. However, the pathogenic role of HMGB1 and the effect of its inhibitor, EP, in pathological retinal neovascularization have remained uncertain. In the present study, we examined whether EP has a preventive effect against pathological retinal neovascularization and inhibits HMGB1 expression in a mouse model of oxygen-induced retinopathy (OIR).

## 2. Materials and Methods 

### 2.1. A Mouse Model of Oxygen Induced Retinopathy

OIR was induced in mouse (C57BL/6) pups according to Smith et al. [18] with some modifications [19]. Briefly, postnatal day 7 (P7) mice, with their nursing mothers, were exposed to hyperoxia (75 ± 2% oxygen) for five days (P7–P11) to produce retinal vasoobliteration and then returned to normoxia (room air) for five days (P12–P16) to induce ischemic retinal neovascularization. At P12, after the pups were exposed to 75% oxygen, they were randomly assigned to three groups: EP-50 (50 mg/kg/day), EP-100 (100 mg/kg/day), and the OIR. The normal group was maintained in room air from birth until postnatal day 17 (P17). EP (Sigma-Aldrich, St. Louis, MO, USA) was diluted with Hartmann's solution (6.0 g NaCl, 0.3 g KCl, 0.2 g CaCl_2_·2H_2_O, and 3.1 g C_3_H_5_NaO_3_/L, Choongwae Pharma, Republic of Korea). The pups were injected in 100 *μ*L intraperitoneally once a day for five days. In the normal group, Hartmann's solution was injected. At P17, after five days of intraperitoneal injection, the mice were anesthetized and sacrificed. These experiments were repeated four times by four animals in each group. All of the experiments were approved by the Korean Institute of Oriental Medicine Institutional Animal Care and Use Committee.

### 2.2. Fluorescein-Dextran Microscopy

At P17, the mice were deeply anesthetized and then 0.1 mL of phosphate-buffered saline (PBS) containing 5 mg of fluorescein-dextran (FD40S, Sigma, St. Louis, MO, USA) was circulated through the left ventricle. The retinas were dissected, flat mounted onto glass slides, and viewed by fluorescence microscopy (BX51, Olympus, Tokyo, Japan). Quantification of the central nonperfused area of the retina was performed using the ImageJ software (NIH, MD, USA).

### 2.3. Lectin Staining

Flat-mounted retinas were fixed in 4% paraformaldehyde for 3 h at room temperature. The retinas were washed with PBS and then incubated for 3 h on an orbital shaker at room temperature with 5% Triton X-100 and 1% BSA. The retinas were washed with PBS and incubated overnight at 4°C with *Bandeiraea simplicifolia* isolectin B4 (1 : 50, Sigma-Aldrich, St. Louis, MO) diluted in PBS. The retinas were washed with 0.05% Tween 20 in PBS followed by incubation with streptavidin TRITC (1 : 500, Serotec, Oxford, UK) for 4 h at 37°C.

### 2.4. Protein Extraction and Western Blot

Protein was extracted from formalin-fixed, paraffin-embedded retinas. Retinal tissue sections were deparaffinized, hydrated with water, and pelleted. The samples were added in extraction buffer, 20 mM Tris HCl buffers (pH 4) with 2% SDS, incubated on ice for 5 min, and mixed by vortexing then boiled at 100°C for 20 min followed by an optional incubation at 80°C for 2 hours. After protein extraction, any remaining unsolubilized material was pelleted at 14000 ×g for 20 min, and protein concentration of total protein extracted was determined by the BCA Protein Assay (Pierce Chemicals Co., Rockford, IL,USA) [20]. The protein was separated by SDS-polyacrylamide gel electrophoresis and transferred to PVDF membrane (Biorad, CA, USA). Membrane was probed with anti-HMGB1 antibody (Abcam, MA, USA) and anti *β*-actin antibody (Sigma, MO, USA), and then the immune complexes were visualized with an enhanced chemiluminescence detection system (Amersham Bioscience, NJ, USA).

### 2.5. Histopathology and Immunohistochemistry

Mice from all of the groups were sacrificed on P17. The eyes were removed and fixed with Davidson's solution at room temperature for 24 h and embedded in paraffin. The paraffin sections were deparaffinized, hydrated with water, and stained with hematoxylin and eosin. For immunohistochemical HMGB1 staining, paraffin sections were deparaffinized, rinsed with 3% hydrogen peroxide, and boiled in citrate buffer (pH 6.0) for 15 min. These sections were washed with PBS, blocked with CAS-Block (Invitrogen, CA, USA) for 30 min, and subsequently incubated overnight at 4°C with a rabbit anti-HMGB1 antibody (1 : 1000, Epitomics, CA, USA). After washing with PBS, the sections were incubated with polymer peroxidase-conjugated rabbit anti-IgG for 30 min at room temperature and were visualized using a 3,3′-diaminobenzidine tetrahydrochloride (ImmPACT DAB, VECTOR, CA, USA) solution and hematoxylin (VECTOR, CA, USA).

### 2.6. Statistical Analysis

The data were expressed as the mean ± SE and analyzed by one-way analysis of variance (ANOVA) followed by Tukey's multiple comparison test or by an unpaired Student's *t*-test using GraphPad Prism 6.0 software (GraphPad, CA, USA). Differences with a value of *P* < 0.05 were considered statistically significant.

## 3. Results

### 3.1. Retinal Neovascularization and Expression of HMGB1 in the Retina of OIR Mice

**Figure 1:**
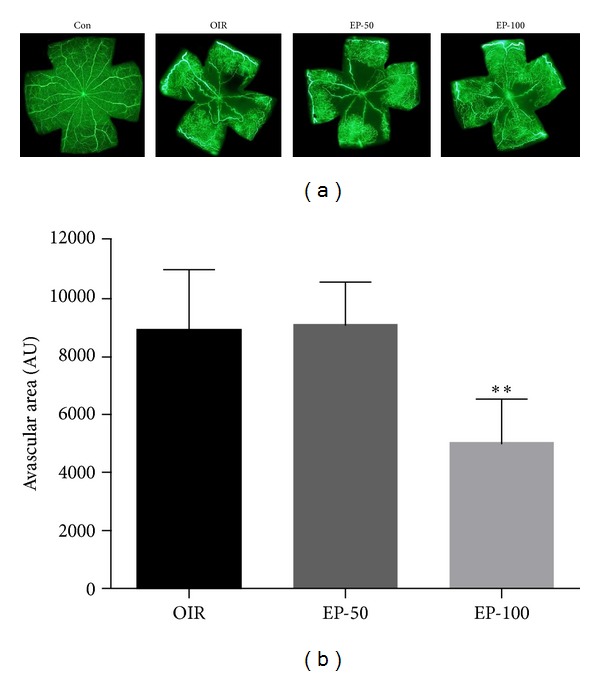
The effect of EP on retinal neovascularization in OIR mice. (a) The retinal vasculature was determined by fluorescein angiography using FITC-dextran. Whole mount retinal preparation from P17 control (Con), OIR (OIR), 50 mg/kg EP-treated (EP-50), and 100 mg/kg EP-treated (EP-100) mice. (b) Quantitative analyses revealed that EP treatment significantly reduced the area of neovascularization in retinas compared with OIR. The data are expressed as the mean ± SE (*n* = 4). **P < 0.01 versus the OIR group.

**Figure 2:**
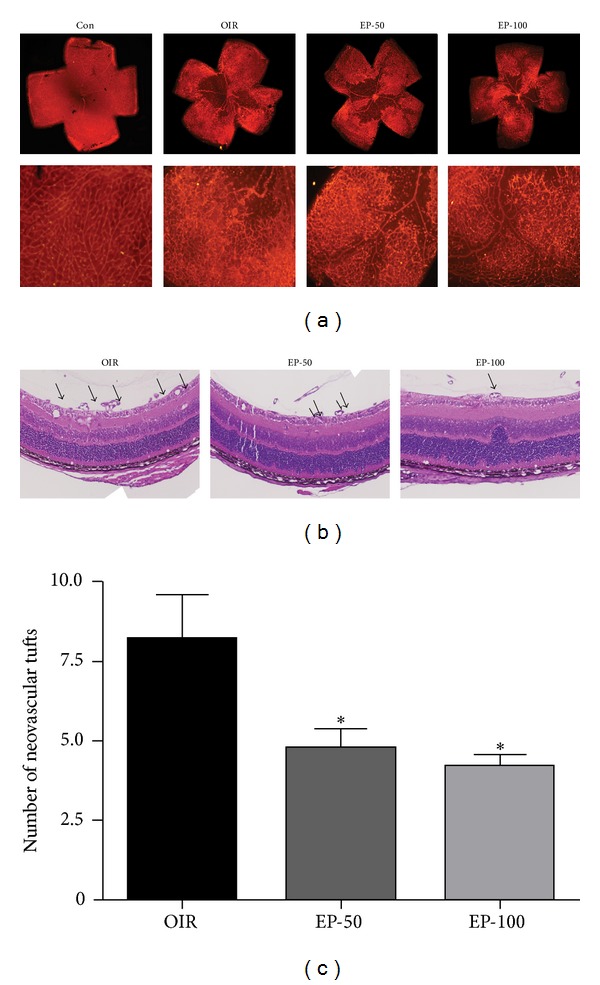
The effect of EP on retinal neovascular tufts in OIR mice. (a) The retinal neovascular tufts were determined using isolectin B4 staining. The retinal preparation from P17 control (Con), OIR (OIR), 50 mg/kg EP-treated (EP-50), and 100 mg/kg EP-treated (EP-100) mice. (b) The arrows indicate neovascular tufts of intravitreous neovascularization. Hematoxylin and eosin-stained cross-section preparations are from P17 OIR (OIR) 50 mg/kg EP-treated (EP-50) and 100 mg/kg EP-treated (EP-100) mice. (c) Quantitative analyses revealed that EP treatment significantly reduced the retinal neovascular tufts compared with OIR. The data are expressed as the mean ± SE (*n* = 4). *P < 0.05 versus the OIR group.

**Figure 3:**
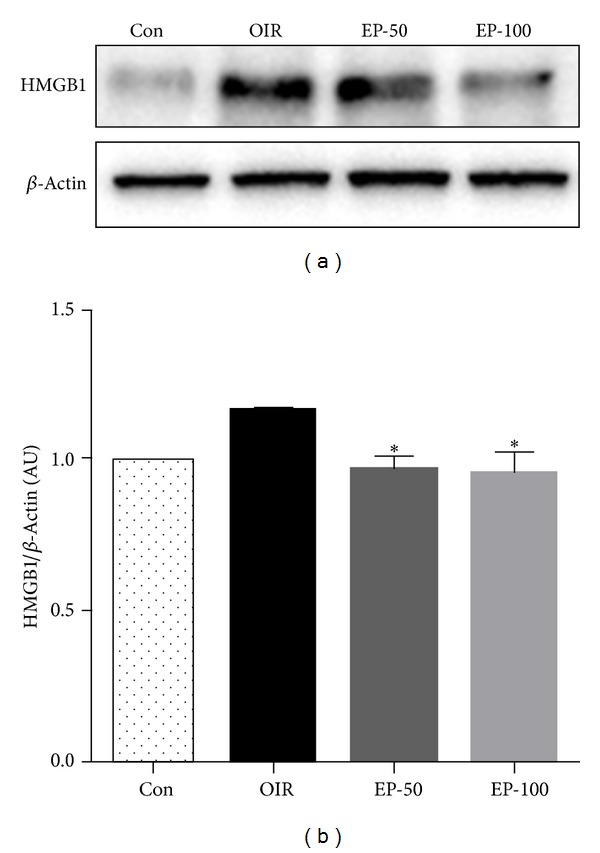
The effect of EP on retinal of HMGB1 expression in OIR mice. Western blot analysis in retina tissue from control (Con), OIR (OIR), 50 mg/kg EP-treated (EP-50), and 100 mg/kg EP-treated (EP-100) mice. The data are expressed as the mean ± SE (*n* = 6). *P < 0.05 versus the OIR group.

**Figure 4:**
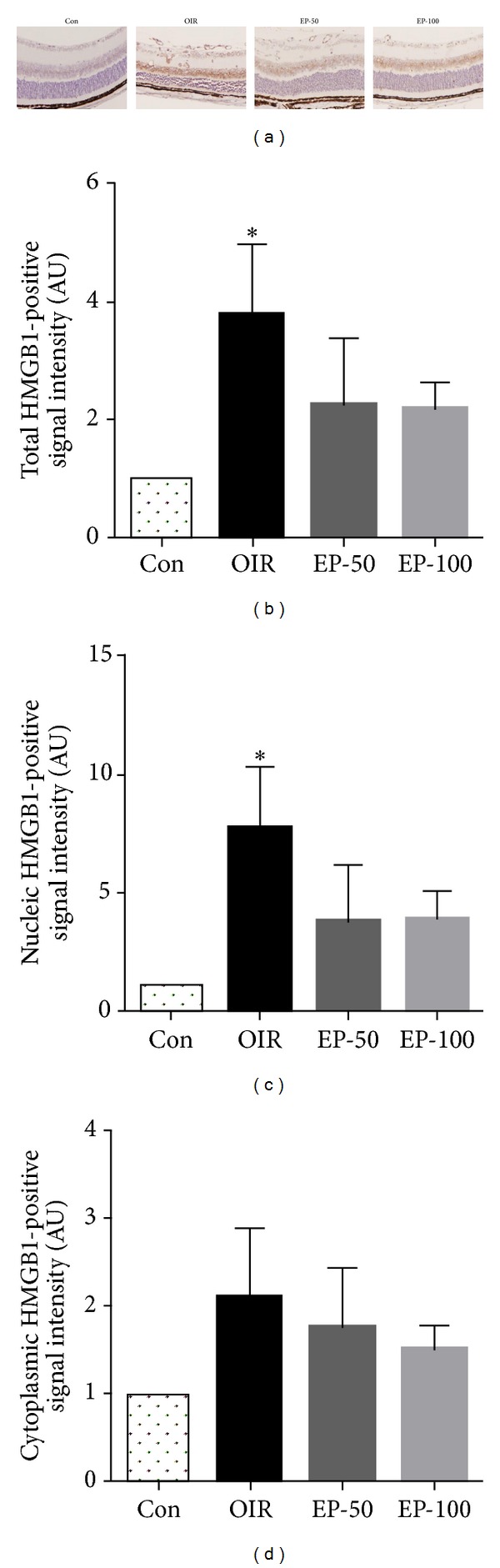
The effect of EP on retinal of HMGB1 expression in OIR mice. (a) Representative photomicrographs of retinal sections labeled with an anti-HMGB1 antibody. Retinas from control (Con), OIR (OIR), 50 mg/kg EP-treated (EP-50), and 100 mg/kg EP-treated (EP-100) mice. (b) Morphological quantitative analyses of nuclei (c) and cytoplasm (d) HMGB1-positive signal intensity. The data are expressed as the mean ± SE (*n* = 6). *P < 0.05 versus the Con group.

HMGB1 has been recognized as a proinflammatory cytokine and more recently as a proangiogenic factor [6, 9]. We therefore determined the expression levels of HMGB1 and its distribution in the retina of OIR mice by immunohistochemistry. First, retinal neovascularization was qualitatively analyzed using fluorescein angiography and quantitatively analyzed by counting neovascular tufts using isolectin B4 and H&E staining. The OIR mice showed a characteristic loss of central retinal vessels by P12, followed by hypoxia-induced regeneration of the central vascular plexus and the development of preretinal neovascularization [18]. In the OIR group, the neovascular response occurred predominantly at the junction between the nonperfused retina and perfused retina. The retinas of OIR mice had an area of multiple neovascular tufts (Figures 1 and 2). In western blot analysis and immunohistochemical staining for HMGB1, we found that HMGB1 was increased in the retinas of OIR mice compared with the control group (Figures 3 and 4). HMGB1 is highly expressed in ganglion cells, inner nuclear layers, outer nuclear layer, and retinal vasculatures, and HMGB1 levels were higher in the inner nuclear layer than the outer nuclear layer in OIR mice (Figure 4). Elevated expression of HMGB1 was detected in the nuclei as well as in the cytoplasm in OIR mice. These results suggest that HMGB1 is highly produced and translocates into the cytoplasm in retina of OIR mice. It was previously reported that a dramatic alteration of retinal gene expression occurred in OIR mice [21], and a high level of retinal HMGB1 expression might be related to active gene transcription [22]. Thus, an increase in HMGB1 might be related to active angiogenic-related gene transcription. 

### 3.2. The Effect of EP on Retinal Neovascularization and HMGB1 Expression in OIR Mice

EP is a potent HMGB1 inhibitor [15]. To further investigate the proangiogenic role of HMGB1, we examined whether EP has a preventive effect against pathological retinal neovascularization and inhibits HMGB1 expression. To test whether pharmacological inhibition of HMGB1 decreases pathological retinal neovascularization, the mice were injected intraperitoneally with EP from P12 to P16. The OIR mice induced the formation of neovascular tufts that penetrate the internal limiting membrane of the retina (Figure 2). Five days after the injection (at P17), there were dose-dependently fewer neovascular tufts in the EP-injected eyes compared to the OIR group (Figure 4, *P* < 0.05). Moreover, the treatment with EP significantly reduced retinal HMGB1 expression in OIR mice (Figures 3 and 4). It thus seemed that HMGB1 in the retina of OIR mice has a proangiogenic role and EP has an antiangiogenic effect via inhibition of the production and release of HMGB1. In addition, the retinas of OIR mice had an area of nonperfusion in the center, tortuous vessels (Figure 1). However, the EP (100 mg/kg)-treated mice had a significantly smaller nonperfused area compared to controls (Figure 1, *P* < 0.01). This result suggests that EP treatment elicits retinal revascularization in OIR mice.

## 4. Discussion 

Retinal neovascularization (RNV) is a major cause of blindness associated with ischemic retinopathy [2, 23]. OIR is a well-established animal model of proliferative ischemic retinopathy. In the OIR model, revascularization of the vasoobliteration (VO) area has progressed farther and pathologic neovascularization formation is at its maximum at P17. The VO area is then fully revascularized in all of the layers, and neovascularization is completely resolved at P25 [24]. In this study, to verify the pathogenic role of HMGB1 in retinal neovascularization, we determined the expression levels of HMGB1 and its distribution in the retina of OIR mice and examined whether EP, a well-known HMGB1 inhibitor, could prevent this retinal neovascularization in OIR mice. We showed that nuclear and cytoplasmic overexpression of HMGB1 and subsequent angiogenesis occurred in OIR mice. However, the treatment with EP dramatically promoted stable vascular growth and blocked retinal pathologic neovascularization. Based on these results, it is likely that the retinal pathogenic angiogenesis that occurs in ischemic retinopathy could also be linked to increases in HMGB1 expression. 

Several angiogenic growth factors and cytokines are involved in the pathogenesis of retinal neovascularization [25–27]. A critical role for HMGB1 in angiogenesis has been recently suggested by Wake et al. who reported that histidine-rich glycoproteins block HMGB1-heparin complex-induced vessel sprouting in Matrigel plugs [28]. HMGB1 signals through the RAGE leading to activation of the transcription fact or nuclear factor kappa B (NF-*κ*B) and induces the expression of various angiogenic factors [9, 29]. HMGB1 and RAGE are expressed by vascular endothelial cells [30]. HMGB1 administration significantly increases the levels of growth factors, including VEGF, released by cultured human cardiac fibroblasts [31]. Although concrete evidence of HMGB1-induced retinal angiogenesis is lacking, our findings suggest that HMGB1 might provide the mechanistic link between ischemic retinopathy and pathogenic angiogenesis. 

In OIR, ROS are increased in the retina and nicotinamide adenine dinucleotide phosphate (NADPH) oxidase is activated, causing apoptosis in endothelial cells, which contributes to avascular retinas [32]. NADPH oxidase activation is exacerbated, contributing to angiogenic blood vessel growth into the vitreous [33]. ROS are increased in the retina, which directly correlates with VEGF expression and angiogenesis [34]. In addition, oxidative stress in various cells is induced in ischemic injury, and then HMGB1 is actively secreted into the extracellular environment under these conditions [35]. Hydrogen peroxide (H_2_O_2_) and the loss of superoxide dismutase 1 (SOD1) mediated oxidative stress promote cytosolic HMGB1 expression and extracellular release [36]. Although we did not test the retinal oxidative status in our OIR mice, these findings suggest that increased retinal HMGB1 expression might be mediated through retinal ROS generation.

In this study, we showed that EP treatment prevents the overexpression of nuclear and cytoplasmic HMGB1 and retinal pathogenic angiogenesis in OIR mice. EP has salutary effects in lethal sepsis and systemic inflammation [15], hemorrhagic shock [37], and stroke [38] and pressure-induced retinal damage [39] and ameliorates murine colitis [40] and renal ischemia and reperfusion injury [41]. The pharmacological effects of EP include amelioration of redox-mediated damage to cells and tissues and the scavenging of ROS [16, 42]. EP regulates the activation of the expression of inflammatory proteins such as IL-6 and TNF-*α* and the inflammatory transcription factor NF-*κ*B [43, 44]. Recently, it was reported that EP has anti-HMGB1 activity. This pharmacological agent has been shown to interfere specifically with HMGB1 released from the nucleus into the extracellular space [15]. In a previous study, EP prevented the increase of HMGB1 mRNA expression after RAW264.7 cells were stimulated with LPS [45]. Although EP is expected to exert an antioxidant effect, EP could be a potent HMGB1 inhibitor. This observation suggests that the antiangiogenic effect of EP is attributable, at least in part, to not only its antioxidative but also HMGB1-inhibitory properties. Therefore, to confirm a proangiogenic role of HMGB1, we also performed a similar animal study using a selective inhibitor of HMGB1, glycyrrhizin (GL). GL is a triterpenoid saponin glycoside of glycyrrhizic acid and its mechanism of action is different from EP. EP inhibits the expression and cytoplasmic release of HMGB1 but do not bind directly to HMGB1 and thus cannot block its extracellular functions. In contrast, GL does not interfere with HMGB1 release, but directly inhibit its extracellular cytokine activities [46]. GL treatment also significantly reduced the area of neovascularization in retinas compared with OIR (see Supplementary data in Supplementary Material available online at http://dx.doi.org/10.1155/2013/245271). This observation also strongly supports the proangiogenic role of HMGB1 in OIR mice.

In conclusion, our study shows for the first time that EP inhibits the retinal pathogenic angiogenesis induced by ischemic retinopathy in OIR mice. In addition, HMGB1 overexpression induced by ischemic retinopathy was significantly inhibited by treatment with EP. These observations suggest that EP acts through an anti-HMGB1 mechanism to protect against retinal pathogenic angiogenesis. Taken together, these results indicate that treatment with EP could be a valuable therapeutic approach in the treatment or prevention of ischemic retinopathy. 

## Supplementary Material

We performed animal study using a selective inhibitor of HMGB1, glycyrrhizin (GL). GL is a triterpenoid saponin glycoside of glycyrrhizic acid. GL does not interfere with HMGB1 release, but directly inhibit its extracellular cytokine activities [46]. GL treatment also significantly reduced the area of neovascularization in retinas compared with OIR.
