# Supplementary material for: Ethyl Pyruvate Inhibits Retinal Pathogenic Neovascularization by Downregulating HMGB1 Expression
Source: J Diabetes Res. 2013 Nov 25;2013:245271. doi: 10.1155/2013/245271 (PMC3858882; doi:10.1155/2013/245271)

Electronical Supplemental Materials

ESM Methods

A mouse model of oxygen induced retinopathy

OIR was induced in mouse (C57BL/6) pups according to Smith et al. [18] with some modifications [19]. Briefly, postnatal day 7 (P7) mice, with their nursing mothers, were exposed to hyperoxia (75±2% oxygen) for five days (P7-P11) to produce retinal vaso-obliteration and then returned to normoxia (room air) for five days (P12-P16) to induce ischemic retinal neovascularization. At P12, after the pups were exposed to 75% oxygen, they were randomly assigned to two groups: Glycyrrhizine (GL)-10 (10 mg/kg/day), and the OIR group. The normal group was maintained in room air from birth until postnatal day 17 (P17). Glycyrrhizine (Wako, Pure Chem. Ind., Osaka, Japan) was dissolved in dimethyl sulfoxide (DMSO) and diluted with Hartmann's solution (6.0g NaCl, 0.3g KCl, 0.2g $CaCl_2 \cdot 2H_2O$ and 3.1g $C_3H_5NaO_3$ /L, Choongwae Pharma, Korea) to adjust the final concentration of DMSO to 5%. The pups were injected intraperitoneally with 100ul once a day for five days. In the normal group, 5% DMSO with Hartmann's solution was injected. At P17, after five days of intraperitoneal injection, the mice were anesthetized and sacrificed. These experiments were repeated four times by four animals in each group. All of the experiments were approved by the Korean Institute of Oriental Medicine Institutional Animal Care and Use Committee.

Fluorescein-Dextran Microscopy.

At P17, the mice were deeply anesthetized and then 0.1 ml of phosphate-buffered saline (PBS) containing 5 mg of fluorescein-dextran (FD40S, Sigma, St. Louis, MO, USA) was circulated through the left ventricle. The retinas were dissected, flat mounted onto glass slides and viewed by fluorescence microscopy (BX51, Olympus, Tokyo, Japan). Quantification of the central nonperfused area of the retina was performed using the ImageJ software (NIH, MD, USA).

Lectin Staining

Flat-mounted retinas were fixed in 4% paraformaldehyde for 3 h at room temperature. The retinas were washed with PBS and then incubated for 3 h on an orbital shaker at room temperature with 5% Triton X-100 and 1% BSA. The retinas were washed with PBS and incubated overnight at 4℃ with *Bandeiraea simplicifolia* isolectin B4 (1:50, Sigma-Aldrich, St. Louis, MO) diluted in PBS. The retinas were washed with 0.05 % Tween 20 in PBS followed by incubation with streptavidin TRITC (1:500, Serotec, Oxford, UK) for 4 h at 37 °C.

Legends for ESM Figures

ESM Figure 1. The effect of GL on retinal neovascularization and retinal neovascular tufts in OIR mice. (A) The retinal vasculature was determined by fluorescein angiography using FITC-dextran. Whole mount retinal preparation from P17 control (Con), OIR (OIR) and 10 mg/kg GL-treated (GL-10) mice. (B) The retinal neovascular tufts were determined using isolectin B4 staining. The retinal preparation from P17 control (Con), OIR (OIR) and 10 mg/kg GL-treated (GL-10) mice. (C) Quantitative analyses revealed that GL treatment significantly reduced the area of neovascularization in retinas compared with OIR. The data are expressed as the mean ± SE (n = 4).

Results

we performed animal study using a selective inhibitor of HMGB1, glycyrrhizin (GL). GL is a triterpenoid saponin glycoside of glycyrrhizic acid. GL does not interfere with HMGB1 release, but directly inhibit its extracellular cytokine activities [46]. GL treatment also significantly reduced the area of neovascularization in retinas compared with OIR.

ESM Figure 1

Figure 2.

(A)

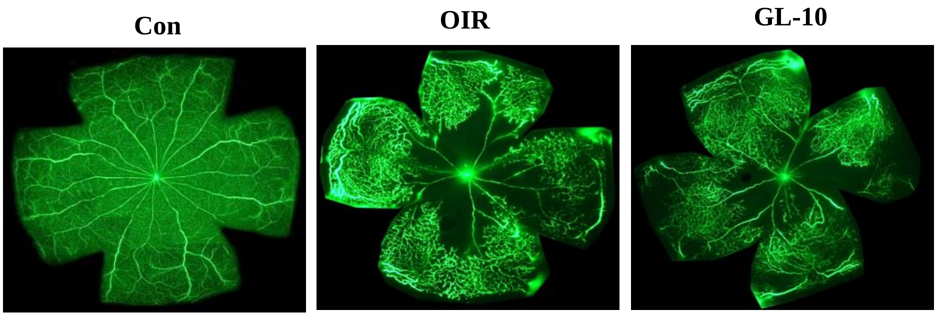

(B)

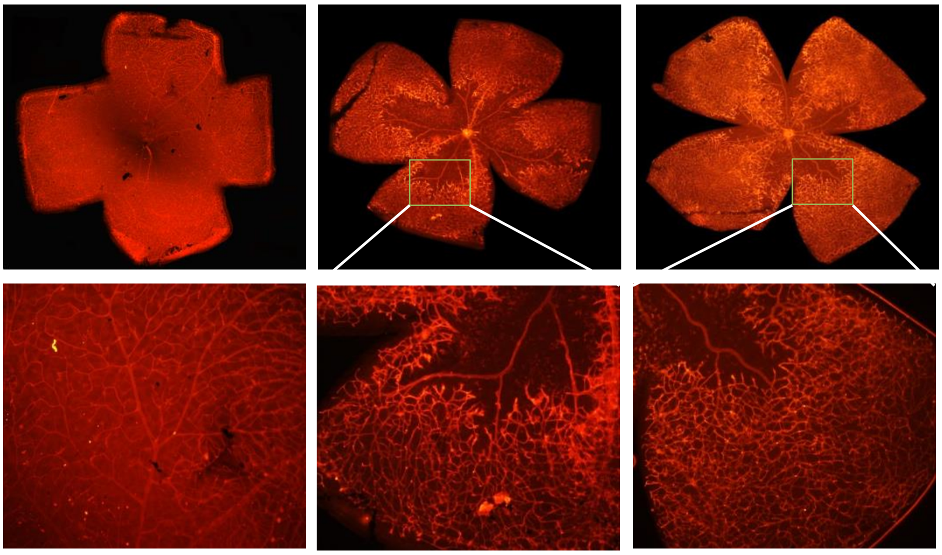

(C)

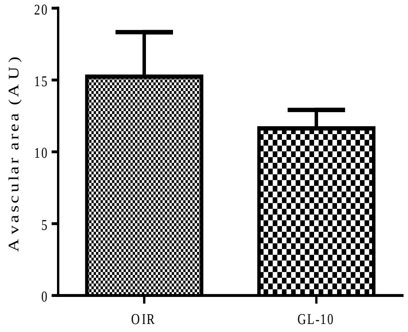

Supplement: Supplementary file 1 — We performed animal study using a selective inhibitor of HMGB1, glycyrrhizin (GL). GL is a triterpenoid saponin glycoside of glycyrrhizic acid. GL does not interfere with HMGB1 release, but directly inhibit its extracellular cytokine activities [46]. GL treatment also significantly reduced the area of neovascularization in retinas compared with OIR. [file 245271.f1.pdf]
